# Effects of phosalone consumption via feeding with or without sodium bentonite on performance, blood metabolites and its transition to milk of Iranian Baluchi sheep

**DOI:** 10.1186/s40781-017-0135-7

**Published:** 2017-05-15

**Authors:** Mohsen Kazemi, Ameneh Eskandary Torbaghan, Abdoul Mansour Tahmasbi, Reza Valizadeh, Abbas Ali Naserian

**Affiliations:** 1 Department of Animal Science, Faculty of Agriculture and Animal Science, University of Torbat-e Jam, Torbat-e Jam, Iran; 2 Department of Environmental Health Engineering, Torbat-e Jam Faculty of Medical Sciences, Torbat-e Jam, Iran; 3 Department of Animal Science, Faculty of Agriculture, Ferdowsi University of Mashhad, Mashhad, Iran

**Keywords:** Sodium bentonite, Phosalone, Iranian Baluchi sheep, Milk, Performance

## Abstract

**Background:**

Transfer of pesticides from environment to animal products is inevitable, so the purpose of the present work was to evaluate phosalone consumption via feeding with or without sodium bentonite (SB) on performance, blood metabolites and its transition to milk of Iranian Baluchi sheep.

**Methods:**

Twenty Baluchi ewes were divided into four treatments (P1 as control, P2, P3, and P4) of five animals in which phosalone, an organophosphate pesticide, was given via diet (only for P2 and P3) at a dose of 280 mg/sheep/day for 63 consecutive days. The SB (32 g/sheep/day; for P3 and P4) was also evaluated for its ability to reduce deleterious effects of phosalone in the sheep diets. The control group (P1) did not receive any phosalone and SB during the experiment. Sampling was conducted in two periods of time including weeks 5 and 9.

**Results:**

Phosalone residues were observed in the milk samples of P2 and P3 groups during two sampling periods. During period 1, the transfer rate of phosalone from feed to milk was 0.23 and 0.02%, respectively for the contaminated diets (P2 and P3), which is relatively similar to period 2 (0.22 and 0.02%). Only 0.34 (period 1) and 0.36% (period 2) of phosalone residue are excreted in the feces of P2 group following its daily consumption. Transfer of phosalone from feed to milk was affected (*P* < 0.05) by the dietary inclusion of a commercial SB, as it (SB) decreased excretion of phosalone via milk (P3). The phosalone and SB alone or together had no significant effect (*P* > 0.05) on the dry matter intake (DMI) and body weight (BW) gain, but feed efficiency, milk production, milk fat, dry matter (DM) and organic matter (OM) digestibility, acetylcholinesterase (AChE) inhibitory activity, hemoglobin (Hb), red blood cell (RBC), serum glutamic pyruvic transaminase (SGPT), serum glutamic oxaloacetic transaminase (SGOT), albumin and mean corpuscular hemoglobin concentration (MCHC) affected by the treatments in period 1 or 2 (*P* < 0.05). The Hb, RBC, and MCHC were significantly decreased (*P* < 0.05) by about 9.72, 20.77, and 9.71%, respectively in the group P2 as compared to those of the control group during period 1. The AChE inhibitory activity (period 1 and 2) significantly increased when phosalone administered via the diet (*P* < 0.05).

**Conclusions:**

Although there were no adverse effects on the performance of sheep following the intake of phosalone alone (P2 vs. P1), but other research on the long and short times to the phosalone in high and low doses with more animals is suggested. Overall, compared to the control group, addition of SB in the diet of sheep improved nutrient digestibility, animal performance, and milk health.

## Background

Animal feed is sometimes contaminated with pesticide residues [69] and these residues may be passed into the body and animal products [68]. In some cases, residues of pesticides are detoxified by the biological system of the body and excreted in urine and feces [38, 52]. Pesticides are materials with the chemical or biological origin that used extensively by human society and can be extremely toxic and harmful to both people and animals. The hazard of cancer via pesticide exposure have examined by many researchers [33, 62, 89]. In order to control pests in Iran, about 5261.80 tons pesticides are used in 2013 [29]. Phosalone is an organophosphate pesticide (OP) that has been widely used by Iranian farmers. Phosalone residues in food and feed with different concentration have been reported by researchers in Iran and other countries [76, 88, 93]. Low levels of phosalone were found [80] in fat and liver (only at 100 ppm) of beef cattle and sheep when administrated in feed at concentrations of 10, 30, and 100 ppm. No residues of phosalone were observed in the milk of dairy cattle at levels of 25, 50 or 100 ppm [81]. Organophosphate pesticides (OPs) are considered as inhibitors of AChE activity and subsequently, accumulation of excessive acetylcholine occur in synaptic cleft available in any live organisms [19, 52, 73]. The residues of several pesticides such as phosalone (0.29 ng/g) in human breast milk were detected by Sharma et al. [85]. Toxic effects of phosalone administered in the feed of rats (0, 5, 50, or 1000 ppm) were investigated by Barker and Sortwell [9] for two consecutive years, but the concentration of 1000 ppm being reduced to 500 ppm at week 27. The Clinical effects such as abnormal posture, hypersensitivity, depression of AChE activity, and poor grooming were observed in groups exposed to 500 ppm phosalone. The enlargement and foamy change in the zona glomerulosa of adrenal gland were observed in rats exposed to 500 ppm. Intestinal epithelial proliferation was found in the normal rat intestinal cells treated with diazinon [35]. Reddy et al. [77] reported hypoventilation and a hypoxic condition in rats exposed to sublethal doses of phosalone. Many methods have been employed for the removal of pesticides and their destructive effects on the body of animals (e.g. use of activated carbon and phenobarbital feeding by Cook and Wilson, [20]; application of atropine as an antidote against OPs by Proskocil et al. [75]; α-lipoic acid supplementation by Al-Attar, [4]). Bentonite as volcanic clay is being used with different goals in industrial agriculture today. Supplementation of contaminated diets with SB decreased transition of aflatoxin M1 to goat’s Milk [70]. Also bentonite has been used as bleaching [48], remove pathogens from the gastrointestinal of poultry [74], toxin binder [56], stabilization of sewage sludge containing heavy metals [49], buffering agent in feedstuffs [24], manipulation of a rumen ecosystem [39, 67] and performance of broiler chickens [55]. The performance of fattening Zandi lambs improved with the dietary inclusion of SB compared to control group [54] and also, fat-tail percentage decreased. The SB participates by manipulation of volatile fatty acid (VFA) profiles, decreasing the dilution rate and subsequently slowing passage rate, changing of the ion exchange capacity of minerals and inactivation the harmful health effects of mycotoxins [47]. There was very little information about transferring of phosalone from contaminated diets to milk and its impacts on blood metabolites and performance of sheep. Although the effects of SB inclusion in the diet of livestock have been studied enormously, it is unknown whether the SB administration via feeding affect ability of animals for reduction of phosalone residues in Iranian Baluchi sheep’s milk or not, hence the present study was conducted for evaluating the effects of phosalone consumption via feeding with or without SB on performance, blood metabolites and its transition to milk of Iranian Baluchi sheep.

## Methods

### Animals, diet and treatments

This experiment was conducted at the Research Station of Ferdowsi University of Mashhad located in Mashhad-Ghochan road. Twenty Iranian Baluchi ewes (45 ± 2.5 kg BW, first-parity and early lactation) were randomly allocated to four treatments (P1, P2, P3, and P4) of five ewes each, fed a total mixed ration. Phosalone, an OP, was daily sprayed on the feed and fed to the ewes according to the following method: Treatment P1 = control without phosalone and sodium bentonite (SB); Treatment P2 = phosalone administered in a dose of 280 mg/day/ewe; Treatment P3 = phosalone (280 mg/ewe/day) and SB (32 g/ewe/day) fed for 63 consecutive days; Treatment P4 = feeding SB with concentration of 32 g/ewe/day. In this experiment, the level of 280 mg/day/ewe set based on the existing information on the milk of animals at different levels of consumption. We selected the mentioned dose to ensure enough quantity of phosalone residues would reach the milk to evaluate neutralized effect of SB. The SB with a commercial name of Zarin Binder was supplied by the Vivan Company located in Mashhad, Iran. Phosalone with a pure analytical grade (99.3%) was dissolved in acetone and immediately mixed with a small part of the diet. After evaporation of acetone, contaminated diets offered to the animals. The feed container of animals was checked regularly to insure that no residues of contaminated feed remain. To ease the separation of urine and feces, the ewes kept in individual metabolic cages. Animals were housed in an environment protected from wind and rain. The ewes were fed at 07:30 a.m. and 17:30 p.m. with a total mixed ration (Table 1) containing forage and concentrate in a ratio of 65:35. The diet was formulated according to the recommendation of NRC [72]. Following the 14 day adaptation period, animals were randomly allocated to treatments. The ewes were given free access to fresh water and milked twice a day (06:30 a.m. and 16:30 p.m.).

**Table 1:** Ingredients and chemical composition of an experimental diet fed to Iranian Baluchi sheep

	Diet
Ingredients (% DM)	
Barley silage	65
Corn grain, ground	15
Soybean meal	8.5
Wheat bran	10
Mineral-vitamin premix^a^	0.7
Limestone	0.5
Salt	0.3
Chemical composition (% DM)	
DM	45
CP	15.23
ADF	25.34
NDF	43.50
EE	3.52
Ca	0.54
P	0.44
Ash	7.06
ME (Mcal/kg DM)	2.47

^a^Containing (g/kg premix; DM basis): 60,000 IU of vitamin D, 330,000 IU of vitamin A, 1000 IU of vitamin E, 160 g Ca, 85 g P, 63 g Na, 45 g Mg, 2100 mg Zn, 12 mg Se, 1500 mg Mn, 535 mg Cu, 45 mg I

### Animal sampling

Feed intake, ort and milk yield were recorded daily for each animal. Likewise, feed samples were taken weekly for determination of dry matter and chemical composition. Blood samples (weeks 5 and 9) were gathered just 3 h post the morning feeding in heparin tubes via jugular vein (5-10 mL), centrifuged (3000×g, 10 min), and the supernatant containing plasma fluid was drawn into sterile 1.5 mL micro-tubes and then conserved at −80 °C for the further analysis. Also samples of blood without centrifugation were quickly transferred to the laboratory for hematology analysis. Samples of urine and feces for phosalone residues were collected in weeks 5 and 9 (in 5 successive days) following the different feeding treatments. Ewes were also weighted before morning feeding in weeks 0, 5 and 9 for gain increment. During the collection periods (5 consecutive days in weeks 5 and 9), daily samples of feed and feces were regularly collected and then were mixed together (with a specified ratio) and one subsample considered for next analysis. The milk of 5 consecutive days was sampled from ewes in weeks 5 and 9, mixed basis milk yield and a subsample analyzed for chemical composition and phosalone residues.

### Laboratory instructions

The dry matter (DM), crude protein (CP), ether extract (EE), Ca and P contents were assessed by the methods of AOAC [6]. The method of Van Soest et al. [96] was applied for determination of neutral detergent fiber (NDF) and acid detergent fiber (ADF). Milk composition, including protein, fat, lactose, and total solid were measured using a milkoscan analyzer (Foss Electric, Conveyor 4000, Hillerød, Denmark). Dry matter and organic matter digestibility were calculated by measuring their concentrations and the values of acid insoluble ash (AIA) as an internal marker in the samples of feed and faecal [95]. The QuEChERS method [7] followed by gas chromatography–mass spectrometry (GC/MS) with selected ion monitoring (SIM) was applied for the determination of phosalone residues in the faecal, urine, and milk samples. Analysis method for GC/MS included: oven temperature of 200 °C; injection temperature of 250 °C; column flow of 1 mL/min; ion source temperature of 200 °C; interface temperature of 300 °C; carrier gas of helium with purity >99.999%; and mode of splitless. Extracted samples of 1 μL were injected into GC/MS apparatus. The analytical capillary column was an Rxi-1 ms (length: 30 m, internal diameter: 0.25 mm, df: 0.25 μm; manufactured by Restek of USA). The blood urea nitrogen (BUN), glucose, cholesterol, albumin, serum glutamic pyruvic transaminase (SGPT), serum glutamic-oxaloacetic transaminase (SGOT), and total protein were determined using an auto-analyzer (Biosystems A15; 08030 Barcelona, Spain). The AChE activity of plasma was measured by the method of Elman et al. [26] and enzyme inhibitory activity of each sample as percentage was calculated from the slope (plot between absorbance and time) of the line with or without inhibitor [65]. Determination of hemoglobin (Hb), white blood cell (WBC), red blood cells (RBC), packed cell value (PCV), mean corpuscular hemoglobin (MCH), mean corpuscular volume (MCV), and mean corpuscular hemoglobin concentration (MCHC) were performed using an automated hematology analyzer (CellTac α, MEK-6450, Nihon Kohden, Japan).

### Statistical data analysis

All data for each period were separately analyzed with a completely randomized design using PROC GLM of SAS software (Version 9.1, SAS Institute). Each animal was considered as experimental unit. Statistical model was: Y_ij_ = μ + T_i_ + ε_ij_, where Y_ij_ is the dependent variable; μ is the overall mean; T_i_ is the treatment effects; ε_ij_ is the residual error. Differences between treatment means were determined by Duncan’s multiple range test at level of *P* < 0.05.

## Results

### Feed intake and body weight changes

DMI, Feed efficiency, OM or DM digestibility, and weight characteristics of ewes following treated ration consumption in two experimental periods are given in Table 2. During the two experimental periods, DMI was similar between treatments (*P* > 0.05), but treatment containing SB in period 2 (P4) had significantly higher (*P* < 0.05) feed efficiency, DM and OM digestibility compared the control group (P1). During period 1 and 2, the ewes fed SB had a numerically higher BW (*P* > 0.05). Feeding of phosalone to the ewes (P2) caused no significant effect on OM or DM digestibility compared to control group (*P* > 0.05).

**Table 2:** DM intake, Feed efficiency, OM and DM digestibility, and weight characteristics of ewes following treated ration consumption in two experimental periods

Item	Period 1 (week 5)				SEM	Period 2 (week 9)				SEM
	P1	P2	P3	P4		P1	P2	P3	P4	
Feed										
DMI (kg/day)	1.17	1.27	1.20	1.40	0.07	1.32	1.39	1.36	1.40	0.03
DMI (kg DMI/kg BW)	0.027	0.029	0.027	0.031	0.002	0.030	0.031	0.030	0.030	0.0002
DMI (kg DMI/kg MW)	0.070	0.074	0.070	0.081	0.004	0.077	0.080	0.078	0.079	0.002
Feed Efficiency (kg Milk/kg DMI)	0.92	0.83	0.92	0.85	0.08	0.83^ab^	0.74^b^	0.85^ab^	0.98^a^	0.07
DM digestibility (%)	66.25	66.16	67.56	67.86	0.55	66.31^b^	67.07^ab^	67.15^ab^	67.56^a^	0.28
OM digestibility (%)	68.26^ab^	67.60^b^	68.26^ab^	68.71^a^	0.26	67.40^b^	67.94^b^	68.82^a^	68.97^a^	0.29
Weight characteristics										
BW (kg)	43.00	44.42	44.74	44.80	0.87	43.72	45.12	45.58	45.76	0.86
BW gain (kg/day)	0.002	0.016	0.010	0.007	0.004	0.034	0.033	0.040	0.046	0.004

^a,b^Values containing different letters in each row are significantly different (*P* < 0.05)

P1 = control without phosalone and sodium bentonite (SB); P2 = phosalone given at dose of 280 mg/day/ewe; P3 = phosalone (280 mg/ewe/day) and SB (32 g/ewe/day) fed for 63 consecutive days; Treatment P4 = feeding SB with concentration of 32 g/ewe/day

### Milk composition and its yield

Milk composition and lactation yield of ewes fed the experimental treatments in two sampling periods are presented in Table 3. When SB was added to the diets, milk yield as kg/day or 6% FCM increased (*P* < 0.05) during period 2 and the highest value was observed for the ewes fed treatment P4. There was no significant effect on milk yield (kg/day or 6% FCM) between treatments P1 and P2 (*P* > 0.05) during period 2. Supplementation of the diet with SB increased milk fat (*P* < 0.05), but protein, lactose, and total solids not affected by the treatments (period 1 and 2).

**Table 3:** Milk composition and lactation yield of ewes fed the experimental treatments in two sampling periods

Item	Period 1 (week 5)				SEM	Period 2 (week 9)				SEM
	P1	P2	P3	P4		P1	P2	P3	P4	
Yield										
Milk (kg/day)	1.08	1.02	1.20	1.18	0.09	1.09^b^	1.03^b^	1.16^ab^	1.37^a^	0.09
Milk (6% FCM)	0.96	0.93	1.12	1.77	0.09	0.98^b^	0.94^b^	1.07^b^	1.35^a^	0.08
Milk components (%)										
Fat	4.82^c^	5.08^bc^	5.28^b^	5.94^a^	0.09	4.88^c^	5.02^c^	5.18^b^	5.82^a^	0.05
Protein	4.20	3.60	3.85	3.92	0.21	3.64	3.15	3.70	4.07	0.25
Lactose	4.83	5.05	4.69	4.64	0.28	5.06	4.84	4.90	4.78	0.17
Total solids	14.37	13.69	13.86	14.35	0.48	13.70	12.79	14.10	13.91	0.34

^a, b, c^Values containing different letters in each row are significantly different (*P* < 0.05)

P1 = control without phosalone and sodium bentonite (SB); P2 = phosalone was given at a dose of 280 mg/day/ewe; P3 = phosalone (280 mg/ewe/day) and SB (32 g/ewe/day) fed for 63 consecutive days; Treatment P4 = feeding SB with concentration of 32 g/ewe/day

6% FCM was calculated by this formula = M (0.453 + 0.0912f) [60], that M is the yield of milk (kg), and f is fat percentage of milk

### Hematology and plasma metabolites

Hematology and plasma metabolites of ewes fed with the experimental treatments in two sampling periods are shown in Table 4. Plasma AChE inhibitory activity was significantly higher (*P* < 0.05) in the ewes fed phosalone alone (P2) compared with control group, but glucose, BUN, cholesterol, and total protein were not affected by the treatments during period 1 and 2. Also during period 1, SGPT and SGOT were significantly decreased following phosalone administration in the diet compared to control group (*P* < 0.05). The highest albumin was observed in the treatment containing SB. The addition of SB to the diet had no significant effect on Hb, RBC, and MCHC than the control group; but these parameters were decreased following the dietary inclusion of phosalone (P2) during period 1 and 2. The hematological parameters such as WBC, PCV, MCH, and MCV were not influenced by the treatments in this experiment (*P* > 0.05).

**Table 4:** Hematology and plasma metabolites of ewes fed with the experimental treatments in two sampling periods

Item	Period 1 (week 5)				SEM	Period 2 (week 9)				SEM
	P1	P2	P3	P4		P1	P2	P3	P4	
Plasma metabolites										
Glucose (mg/dl)	57.56	60.48	62.56	61.24	1.75	65.48	66.08	67.10	65.78	0.73
BUN (mg/dl)	21.84	21.66	21.12	20.93	0.41	19.58	20.52	19.62	19.34	0.41
Cholesterol (mg/dl)	76.20	76.36	71.00	73.66	1.67	75.40	79.20	78.74	77.50	2.16
Albumin (mg/dl)	2.92	3.01	3.19	3.06	0.09	3.04^ab^	2.83^b^	3.15^a, b^	3.24^a^	0.12
SGPT (U/L)	23.58	25.60	24.00	25.26	1.86	25.06^b^	29.62^a^	24.84^b^	23.56^b^	1.06
SGOT (U/L)	148.60	160.00	151.80	159.80	4.24	144.92^b^	159.20^a^	151.88^ab^	158.04^ab^	4.31
AChE inhibitory activity (%)	9.40^b^	11.00^a^	9.80^ab^	9.60^ab^	0.45	9.50^b^	11.30^a^	9.50^b^	9.40^b^	0.41
Total Protein (g/dl)	6.32	6.44	6.56	6.54	0.21	6.60	6.57	6.76	7.08	0.16
Hematology										
Hb (g/dl)	9.77^a^	8.82^b^	9.30^ab^	9.56^ab^	0.28	10.10^a^	9.14^b^	10.40^a^	10.04^ab^	0.30
RBC (*10^6^/μl)	10.06^a^	7.97^b^	9.78^a^	9.56^a^	0.44	8.90^ab^	8.64^ab^	8.39^b^	9.51^a^	0.31
WBC (Cell/μl)	6520	7190	6900	6950	307.10	6920	7080	7000	6380	276.11
PCV (%)	30.80	27.80	29.80	30	1.24	30.60	31.20	30.80	31.00	1.02
MCH (pg)	9.78	10.22	9.55	9.67	0.43	11.42	11.52	12.41	11.05	0.46
MCV (fL)	43.59	40.94	41.83	42.22	0.90	38.22	36.22	38.22	37.63	0.75
MCHC (g/dl)	37.57^a^	33.92^b^	35.77^ab^	36.76^ab^	1.09	32.98^ab^	29.44^b^	34.05^a^	32.40^ab^	1.22

^a, b^Values containing different letters in each row are significantly different (*P* < 0.05)

P1 = control without phosalone and sodium bentonite (SB); P2 = phosalone was given at a dose of 280 mg/day/ewe; P3 = phosalone (280 mg/ewe/day) and SB (32 g/ewe/day) fed for 63 consecutive days; Treatment P4 = feeding SB with concentration of 32 g/ewe/day

### Phosalone residues

Monitoring the phosalone residues in ewes fed by the experimental treatments in two sampling periods are presented in Table 5. There was no phosalone residue in treatments P1 and P4, so they withdrew from the statistical analysis. Residues of phosalone were detected in the samples of milk, faecal and urine of both P2 and P3 treatments during period 1 and 2. The addition of SB in the diet decreased transfer rate of phosalone to milk (*P* < 0.05).

**Table 5:** Monitoring the phosalone residues in ewes fed the experimental treatments in two sampling periods

Item	Period 1 (week 5)		SEM	Period 2 (week 9)		SEM
	P2	P3		P2	P3	
Residues (mg/kg milk)	0.630^a^	0.055^b^	0.027	0.608^a^	0.054^b^	0.029
Residues (mg/total milk production/day)	0.641^a^	0.066^b^	0.032	0.623^a^	0.062^b^	0.029
Transfer rate to Milk (%)	0.23^a^	0.02^b^	0.011	0.22^a^	0.02^b^	0.010
Residues in faecal (mg/kg DM)	2.210^b^	2.340^a^	0.007	2.204^b^	2.334^a^	0.006
Residues in faecal (mg/total faecal excretion/day)	0.945	0.913	0.067	1.009	1.046	0.025
Resides in urine (mg/L)	0.190^b^	0.320^a^	0.009	0.196^b^	0.318^a^	0.011

^a, b^Values containing different letters in each row are significantly different (*P* < 0.05)

P2 = phosalone administered in dose of 280 mg/day/ewe; P3 = phosalone (280 mg/ewe/day) and SB (32 g/ewe/day) fed for 63 consecutive days

Phosalone residues not detected in treatment P1 and P4, so they withdrew from the statistical analysis

## Discussion

### Feed intake and body weight changes

The DMI and body weight of lactating crossbred goats were not affected due to feeding monocrotophos at a dose of 25 mg/kg (DM basis) [57]. In the present work treatment of diet with the phosalone had no effect on BW of ewes compared to the control group during two sampling periods. In studies of Sangha et al. [82], feed intake and water intake were affected by cypermethrin at a dose of 50 mg/kg BW in treated rats [82], also the body weight of rats decreased significantly compared to the control group (*P* < 0.05). In this study, supplementing the diet of ewes with SB increased OM and DM digestibility compared with the control group (*P* < 0.05) during period 2. SB is classified as expanded lattice clay in a montmorillonite group of minerals [10]. Having a high potential for ion exchanging in the bentonite can bind a different range of cations [31]. Wool growth improved by supplementation of SB in the sheep diets [17, 30, 31]. Also, SB reduced the concentration of ruminal ammonia and improved the passage of feed and microbial protein to the small intestine [42]. The feed intake and average daily gain of lambs were increased (*P* < 0.05) following SB supplementation [98]. In our study, the average DMI of periods 1 and 2 was 1.24, 1.33, 1.28 and 1.40 Kg/day for the P1, P2, P3, and P4 treatments, respectively, but the differences between treatments were not significant (Table 2). Similarly, feed intake expressed as kg/kg BW, as well as kg/kg MW was not significant (*P* > 0.05). In a study of Berthiaume et al. [11], although supplementation of direct-cut grass silage with bentonite increased the BW gain of steers (*P* < 0.05), it had no effect on DMI and nutrient (DM, OM, NDF, ADF, and nitrogen) digestibility of grass silage. It was found that ciliate protozoa could not consume bentonite interring the rumen; however bentonite interfered with movement efficiency of cilia and subsequently prevented the motility of protozoa, which diminished the power of predation for rumen bacteria [97]. Population of protozoal in ruminal fluid of faunated rams was lower for animals fed bentonite or monensin than to control group [40]. In this experiment the BW gain (kg/day) was comparable between treatments, ranging between 0.018, 0.024, 0.025 and 0.026 kg/day (average period 1 and 2) respectively in P1, P2, P3 and P4 treatments. An increasing trend for DMI and BW gain was observed in the treatments containing SB. The nitrogen utilization was improved due to the feeding of soybean meal with bentonite [16]. The growth performance of lambs was also improved when fed a diet containing 1% of SB [98], but supplementation of a diet with 2.5% decreased the performance of finishing steers [18]. The VFA, BW, DMI, faecal excretion, apparent digestibility of DM, OM, CP, NDF, and ADF were not affected by the 1% SB in the diet of growing lambs compared to the control group [3].

### Milk composition and its production

Due to increase of DMI in the diet containing SB, there was a trend to increase undegradable intake protein (UIP) in the lambs [98]. The increased passage of microbial protein and feed to the small intestine occurred when SB was used [41]. In our experiment (period 2), an increase in milk production may be related to feed improvement and microbial protein supply to the small intestine following SB consumption. It is predicted that higher more bypass protein from the rumen to the small intestine will increase ruminant performance, especially for the meat and milk containing more protein composition. Although SB is suggested to change ruminal microbial populations, but there was observed only an increasing in total VFA (*P* < 0.05) concentrations [98]. In one study conducted with Colling et al. [18], they found an increase in acetate and butyrate concentrations and a decrease in propionate following SB supplementation [18]. In the current research supplementation of the diet with 280 mg of phosalone/day/ewe (P2) had no significant effect on milk yield and milk components (Table 3) compared to the control group (P1), but diet containing SB had a higher milk yield and milk fat during period 2. The application of bentonite in the sorghum grain-based diets did not change the milk composition or production in Holstein–Friesian dairy cows, but the rumen pH increased (*P* < 0.05), faecal starch and rumen ammonia decreased and animals tended to intake less grain sorghum [25]. Similarly, it has been observed that dietary supplements of bentonite diminished microbial degradability of feed protein and increased microbial protein synthesis in the rumen, the small intestine flow of amino acids, and growth of wool in sheep containing regular ruminal microbial population [41]. Although bentonite in P3 could greatly reduce the adverse effects of phosalone in the animals, part of toxicity effects on animal metabolism following excessive intake of phosalone still seems to remain. So in the present study, SB alone (P4) increased the productivity of ewes (i.e., milk yield and milk fat), but phosalone with SB (P3) did not affect those compared with control group during sampling periods.

### Hematology and plasma metabolites

The addition of chlorpyriphos in the diet of calves increased (*P* < 0.05) glucose, ALT, and AST and decreased (*P* < 0.05) the AChE activity [84]. The carp fish were exposed to low (0.15 mg/L), medium (0.3 mg/L), and high (0.6 mg/L) levels of phosalone for 14 consecutive days. The RBC, WBC, hematocrit, Hb, MCV, MCH, and MCHC were significantly affected by levels of phosalone [51]. Oral administration of diazinon at a level of 50 mg/kg BW for 21 days did not significantly accompanied with pathological and clinical symptoms following decreasing the AChE activity in the sheep [5]. In this study, no sign of toxicity was observed until the end of the experiment. The Activity of AChE in the erythrocytes, plasma or serum of sheep after dipping in the diazinon soluble was not affected by pesticide [37]. The AChE is an enzyme which breaks down the acetylcholine into acetate and choline; hence OP can block the activity of this enzyme [36]. The OP compounds inhibit AChE which hydrolyses acetylcholine. Binding of OP with AChE caused to phosphorylation of the enzyme and this reaction is not quickly reversible [34]. Also, it is reported that the toxic effect of organophosphates is caused by phosphorylation of AChE in erythrocytes, nerve tissue, serum, and liver [13]. Use of 1 mL of corn oil containing 100 mg malathion/kg BW of rat per day had lower AChE activity than the control group [79]. Rezg et al. [79] found a marked increase in Hb concentration which can be considered as an adaptive method in order to supply more oxygen in response to pulmonary hurt induced by subchronic exposure to malathion that is inconsistent with our reports. The RBC, Hb, MCV, and MCHC were similar in rats fed with 60 mg sumithion/kg BW/day compared to the control group without pesticide. Anemia was observed about 1 month after administration of terbufos in the cattle [14]. It is reported that malathion caused severe hepatic and renal damages which increased levels of liver enzymes [SGOT, SGPT, alkaline phosphatase (ALP) and acid phosphatase (ACP)] and made many changes in kidney output including significantly increased levels of creatinine, urea and uric acid, and reduced total protein and albumin in the plasma of rats exposed to malathion at a dose level of 20 mg/kg BW [4]. Increasing of SGPT and SGOT [4] is consistent with our experiment after phosalone administration. In this study, although the level of AChE inhibitory activity (17.99%, mean of period 1 and 2) was markedly increased some blood indices such as Hb (9.66%, mean of period 1 and 2), RBC (12.45%), and MCHC (10.2%) were significantly decreased in ewes exposed to phosalone compared to the control group. These results are in line with different previous studies which indicated that the exposure to malathion and other pesticides cause to impel acute physiological and biochemical disorders in experimental animals, buffalo calves [86], goats [50], mice [63], cockerels [87], poultry [43], rabbits [101], and rats [2, 42]. Some of the harmful impacts of oxidative stress leading to the production of free radicals are due to the activity of a number of pesticides known as OP having a tendency to produce of oxidative stress [4]. Nevertheless, several studies showed that malathion impelled lipid peroxidation and oxidative stress in experimental animals [22, 32]. Dimethoate pesticide at a dose of 75 mg/kg BW inhibited AChE activity in Albino rat males [8]. A dose of 0.1 mg malathion/mouse caused a significant decline in RBC, leukocyte, Hb, neutrophil, eosinophil and monocytes contents, and an increase in reticulocytes [12]. Similar changes in RBC and Hb [92] have been found using different pesticides and various experimental methods. In the present study among measured plasma metabolites, AChE inhibitory activity, SGPT, and SGOT is affected by the phosalone feeding. The Hb, RBC, and MCHC of hematology parameters were also decreased with the addition of phosalone to the diet. AChE activity in other studies of human [78], carp [90], mice [59] exposed to organophosphate pesticides was significantly lower than the control group. Levels of blood AChE were markedly depressed only in the animals fed on silage from corn treated at a level of 907 g/0.4 ha, and the common health of the animals and their milk production did not seem to be affected by pesticide [15]. In comparison with other adsorbents to binding toxins [94], it is concluded that bentonites have unique characteristics followed by kaolin-pectin in affinity of toxins (Kaopectate). There was no significant effect on glucose, beta globulin, cholesterol, and total protein, but alpha globulin, gamma globulin, albumin and urea were affected by the treatment of 2% bentonite rather than control group [54]. Despite an increase in albumin (present study), an increase in total protein and a decrease in BUN of plasma can be related to the fact that bentonite application can form a steady state in the rumen following the absorption of ammonia, which alternatively ammonia will be released after the decrease in the pool of the rumen, hence the animals will have a more chance in microbial protein synthesis in the rumen [54]. In the present work, all of the hematology parameters and plasma metabolites not affected by SB supplementation was compared to the control group (*P* < 0.05).

### Phosalone residues

Phosalone residue (mg/kg/day) in the milk of P2 treatment was approximately 11.45 and 11.26 (period 1 and 2, respectively) times more than P3 group. During period 1 and 2, excretion of phosalone via urine and faecal (mg/kg DM) for P3 group was higher than P2. The phosalone excreted via the urine and faecal (mg/kg DM) in the P3 group was approximately 1.65 and 1.06 (period 1 and 2) times more than P2 group, respectively. Fenthion at low levels (0.006-0.014 m/kg) was found only in the milk of cows fed on contaminated corn silage. The residues were also found in the urine (0.004-0.160 ppm) and feces (0.003-0.156 mg/kg, wet basis) of all cows following consuming of fenthion in the diets [15]. In this study, only 0.34 (period 1) and 0.36% (period 2) of phosalone residue is found in the feces of the P2 group following daily consumption of it. The concentration of phosalone in the milk of ewes was (0.630 and 0.608 vs. 0.055 and 0.054 mg/kg for P2 vs. P3 in period 1 and 2, respectively) above the maximum residue limit (MRL) of 0.01 mg/kg suggested by the European Union [27]. Therefore, use of ewe’s milk by humans can have negative effects on their health in the future. The JMPR [44] established an ADI of 0-0.02 mg/kg BW for phosalone. So by considering ADI at 0.02 mg/kg BW, health condition and immune response in persons with 60 kg BW who received 1.2 mg of phosalone/day may not be concerned during a short term. Also the concentration of phosalone in the body of a 60 kg person following consumption 1 kg milk from P2 or P3 treatment will be 0.619 or 0.054 (mean period 1 and 2) mg/day, respectively that both values are less than 1.2 mg, so milk consumption of this study will not be worrying for a 60 kg person in the short term. In Brazil, 30 milk samples and all components of the animals’ diet were tested for pesticide residues. From 30 milk samples, six (20%) were polluted with organophosphates, five (16.7%) with carbamate, and one illustration with both organophosphate and carbamate. From 48 tested feed cases, 15 (31.25%) were polluted to organophosphates, six (12.50%) to carbamate, and one case was contaminated with both pesticides [28]. Phosalone residues and their oxygen analogue were measured in the milk and tissues of dairy cows after administration at doses of 100, 200 and 500 mg/kg diet for 28 days [21]. The maximum phosalone residues with their metabolites at the 100 mg/kg of diet were about 0.03 mg/kg in the fat, 0.3 mg/kg in the liver, 0.05 mg/kg in the kidneys, 0.007 mg/kg in the milk and <0.05 mg/kg in other tissues. Feeding a goat with 957 mg phosalone resulted in excretion of 0.86 mg/kg in the milk, 0.27 mg/kg in the muscle, 1.02 mg/kg in the fat, 12.6 mg/kg in the liver and 13.1 mg/kg in the liver [100]. Chlorpyrifos as an OP fed in dairy cattle ration for 2 consecutive weeks by McKellar et al. [61]. The chlorpyrifos residues and their (mainly oxidized and hydroxylated) metabolites were observed at low doses in milk, and these residues decreased quickly after cessation of application. A similar finding by Johnson et al. [45] was reported. There were no diazinon residues in the milk of dairy cows following its feeding with a protein supplement [58]. Low levels of diazinon (<0.025–1 mg/kg) were found in the milk of cattle and sheep after consumption [71, 91]. Studies about application of SB for adsorption of OPs after administration is scarce, but the adsorption efficiency of SB for other toxins material in different animal discussed extensively {study of improvement of some serum biochemical changes in the broiler chickens administered 2.5 mg aflatoxin/kg diet by Kececi et al. [53]; the use of bentonite in the diet contaminated with aflatoxin, mainly decreased the unfavorable impacts of the aflatoxin in the rat [1]; using the specific clays (such as bentonite) can seriously diminished some of the negative effects associated with administration of aflatoxin to weanling pigs [83]; application of SB at 1.2% of ration showed good potential as aflatoxin binder and contamination of milk decreased up to 61% in dairy cows [23]}. In the present study, the concentration of phosalone in the milk samples of animals decreased during feeding SB in the P3 group, which indicated that it could not pass readily from the feed or digestive tract to the milk. The calcium bentonites considered as adsorbent clay (due to their surface charge and surface area) with high quality made of silicates or aluminosilicates. Results indicated that most of the calcium bentonites will often adsorb up to 100% of their dry weight of water and 80% oil [46, 66]. Toxins can be conducted into the porous structure of clay via electric elementary charges. The rate of adsorption can be affected by variable parameters such as size and the electric charge of the toxin or structure of clay [46]. Furthermore, micro-elements in the diet can be adsorbed by aluminosilicates and have deleterious impacts on the bioavailability of them in the body of animals [64, 99].

## Conclusion

Using phosalone residues in milk may have unfavorable effects on the health of humans. When ewes fed on 280 mg phosalone per day for 63 consecutive days, pesticide residue found in milk, feces, and urine during both sampling periods. Supplementation of SB at 32 g/sheep/day resulted in significant reduction in phosalone content of milk, and increased milk yield and milk fat content. Also excretion of phosalone via urine and faecal significantly increased by application of SB in the ration. The present study indicates that feeding of phosalone to ewes caused a significant increase in AChE inhibitory activity and a significant reduction in RBC, Hb as well as MCHC. Although there was no significant change in DMI and BW gain following the feeding of both phosalone and SB together or alone in the animals, further studies are needed on a large number of animals.

## Abbreviations

AChE: Acetylcholinesterase
ACP: Acid phosphatase
ADF: Acid detergent fiber
ADI: Acceptable daily intake
AIA: Acid insoluble ash
ALP: Alkaline phosphatase
ALT: Alanine transaminase
AST: Aspartate Aminotransferase
BUN: Blood urea nitrogen
BW: Body Weight
Ca: Calcium
CP: Crude protein
df: Film Thickness (μm)
DM: Dry matter
DMI: Dry matter intake
e.g.: For example
EE: Ether extract
g: gram
GC/MS: Gas chromatography–mass spectrometry
Hb: Hemoglobin
Kg: Kilogram
MCH: Mean corpuscular hemoglobin
MCHC: Mean corpuscular hemoglobin concentration
MCV: Mean corpuscular volume
mg: Milligram
min: Minute
mL: Milliliter
mm: Millimeter
MRL: Maximum residue limit
MW: Metabolic weight
NDF: Neutral detergent fiber
ng: Nanogram
OM: Organic matter
OP: Organophosphate pesticide
OPs: Organophosphate pesticides
P: Phosphorus
PCV: Packed cell volume
ppm: Part per milion
RBC: Red blood cell
SB: Sodium bentonite
SGOT: Serum glutamic oxaloacetic transaminase
SGPT: Serum glutamic pyruvic transaminase
SIM: Selected ion monitoring
UIP: Undegradable intake protein
VFA: Volatile fatty acids
Vs: Versus
WBC: White blood cell
WBC: White blood cells
μl: Microliter
